# Deep learning-based detection of acute pancreatitis on abdominal contrast-enhanced CT

**DOI:** 10.1186/s41747-026-00775-2

**Published:** 2026-07-31

**Authors:** Oleksandra Seidel, Maike Theis, Sebastian Nowak, Laura Garajová, Benjamin Wulff, Wolfgang Block, Lukas Müller, Tilmann Emrich, Julian A. Luetkens, Dariusch R. Hadizadeh, Alois M. Sprinkart, Dmitrij Kravchenko

**Affiliations:** 1 Clinic for Diagnostic and Interventional Radiology, University of Bonn, University Hospital Bonn, Bonn, Germany; 2 Clinic for Radiation Therapy and Radiooncology, University of Bonn, University Hospital Bonn, Bonn, Germany; 3 Clinic for Neuroradiology, University of Bonn, University Hospital Bonn, Bonn, Germany; 4 Department of Diagnostic and Interventional Radiology, University Medical Center of the Johannes Gutenberg-University, Mainz, Germany

**Keywords:** Artificial intelligence, Deep learning, Diagnosis (computer-assisted), Pancreatitis, Tomography (x-ray computed)

## Abstract

**Objective:**

We developed and evaluated a deep learning (DL) model for image-based detection of acute pancreatitis (AP) on abdominal contrast-enhanced CT (CECT).

**Methods:**

A total of 552 patients from two university centers (January 2010–January 2026) were included. The internal dataset comprised 207 patients with clinically and radiologically confirmed AP (499 scans) and 250 control patients with suspected AP (368 scans). An independent external validation cohort included 95 patients. Convolutional neural network–based models were trained using monophasic and biphasic CECT data. The final model was evaluated on a 20% patient-level hold-out test set from the internal cohort and on the external cohort. Performance was assessed using the F1 score and area under the receiver operating characteristic curve (AUROC).

**Results:**

A single-input multiphase model incorporating arterial and portal venous phase scans achieved the best performance, with ensembling applied for biphasic studies. On the internal hold-out test set (*n* = 116), the model achieved an F1 score of 0.83 (95% confidence interval 0.75–0.89) and an AUROC of 0.89 (0.82–0.95). Performance remained robust on external validation (*n* = 95), with an AUROC of 0.99 (0.96–1.00) and an F1 score of 0.92 (0.86–0.97).

**Conclusion:**

DL enabled accurate CECT-based identification of AP in this retrospective multicenter cohort, with performance maintained in an independent external dataset. Prospective validation using broader and independently adjudicated clinical populations remains necessary.

**Relevance statement:**

The model showed promising performance for CECT-based acute pancreatitis detection but was not designed or tested as a triage system.

**Key Points:**

- Diagnostic uncertainty in acute pancreatitis often arises from nonspecific abdominal symptoms and inter-reader variability in CECT interpretation.
- The DL model achieved high internal accuracy (AUROC 0.89) and maintained robust performance in an independent external validation cohort (AUROC 0.99).

**Graphical Abstract:**

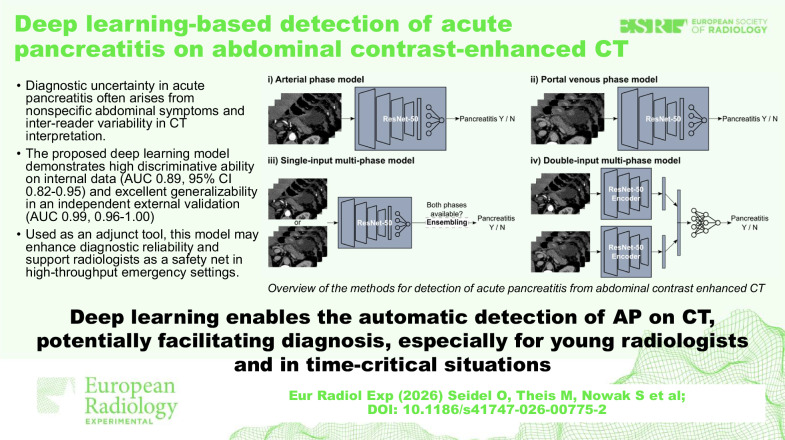

## Background

Acute pancreatitis (AP) is a potentially life-threatening inflammatory disease and remains the most prevalent gastrointestinal disorder requiring immediate hospital treatment and observation. AP poses a significant clinical challenge in both diagnosis and treatment due to its varied clinical and image-based presentation and the risk of severe complications. The global incidence of AP has been rising in recent decades, with regional variations ranging from 13 to 45 cases per 100,000 individuals annually [1–3]. AP ist most commonly caused by excessive alcohol consumption and gallstone-induced obstruction of the pancreatic duct, which together account for 60–80% of cases [4]. Clinical manifestations include a broad spectrum of symptoms, ranging from mild, self-limiting cases to severe, life-threatening forms associated with extensive local and systemic complications, resulting in high morbidity and mortality [5, 6]. Rapid and accurate diagnosis is therefore crucial for appropriate management and prognosis assessment, as the severity strongly influences complications [7, 8].

The diagnostic criteria for AP rely on clinical presentation, biochemical markers, and imaging findings. The diagnosis of AP is based on the fulfillment of two of three criteria: (1) severe upper abdominal pain radiating to the back; (2) serum lipase levels elevated to more than three times the upper limit of normal; and (3) imaging findings consistent with AP (abdominal ultrasound, contrast-enhanced computed tomography (CECT), or magnetic resonance imaging [9–11]). However, severe abdominal pain, often referred to as acute abdomen, can arise from various etiologies, complicating the diagnosis of AP. Additionally, the broad spectrum of symptoms associated with AP and the variability in its presentation can lead to the condition being overlooked, resulting in diagnostic errors and delayed treatment. Careful evaluation of ambiguous cases is essential, as 25% of patients with negative results of serum lipase are incorrectly classified as not having AP, potentially leading to poorer outcomes [12].

Contrast-enhanced CT (CECT) remains the reference standard for confirming AP in uncertain clinical scenarios, narrowing down differential diagnoses, and assessing disease severity [13–17]. While its pivotal role in detecting complications and guiding treatment is well-established, the reliance on human interpretation can be challenged by increasing workloads in emergency settings. Consequently, there is an increasing demand for automated tools to aid in the detection and classification of AP.

The application of artificial intelligence has shown great promise in abdominal imaging [18]. While earlier research primarily focused on predicting severity using clinical scores or laboratory data [19–21], recent studies have begun to address imaging-based detection. For instance, Zhang et al demonstrated that deep learning (DL) models can achieve high diagnostic accuracy in differentiating AP from controls on CECT scans [22].

Despite these promising advancements, a critical gap remains: most existing DL approaches are limited by single-center designs and lack rigorous testing on independent, external cohorts. This limits the generalizability of such tools across different scanners and patient populations, reflecting real-life scenarios. Therefore, the aim of this study was to explore the potential of DL as an image-based second-reader tool for AP detection and, crucially, validate its robustness on an independent external cohort.

## Methods

### Patient characteristics

This retrospective, multicenter study was approved by the local ethics committee of the University of Bonn (application no. 287/23). All examinations were performed in accordance with the Declaration of Helsinki and institutional ethical guidelines. Patients with suspected AP who underwent abdominal CT at the University Hospital of Bonn between January 2010 and August 2023 were considered for inclusion. A full-text search of radiology reports for pancreatitis-related terms was performed to identify relevant cases for the study (Supplementary Material S1). Inclusion in the AP cohort required both imaging findings and clinical AP confirmation in accordance with established diagnostic guidelines [10, 11]. Radiological reports were reviewed to confirm the documented diagnosis based on descriptions of pancreatic edema/enlargement, peripancreatic edema/fluid, or pancreatic/peripancreatic necrosis [9, 23]. Clinical confirmation of AP was verified using International Classification of Diseases, 10th Revision (ICD-10) codes K85 and K86 recorded within 6 months of the CT scan. Detailed information on the ICD-10 codes considered can be found in Supplementary Material S2. The control group included patients presenting with acute abdominal symptoms or clinically suspected but unconfirmed AP, who underwent abdominal CT but showed no radiological signs of AP.

**Fig. 1:**
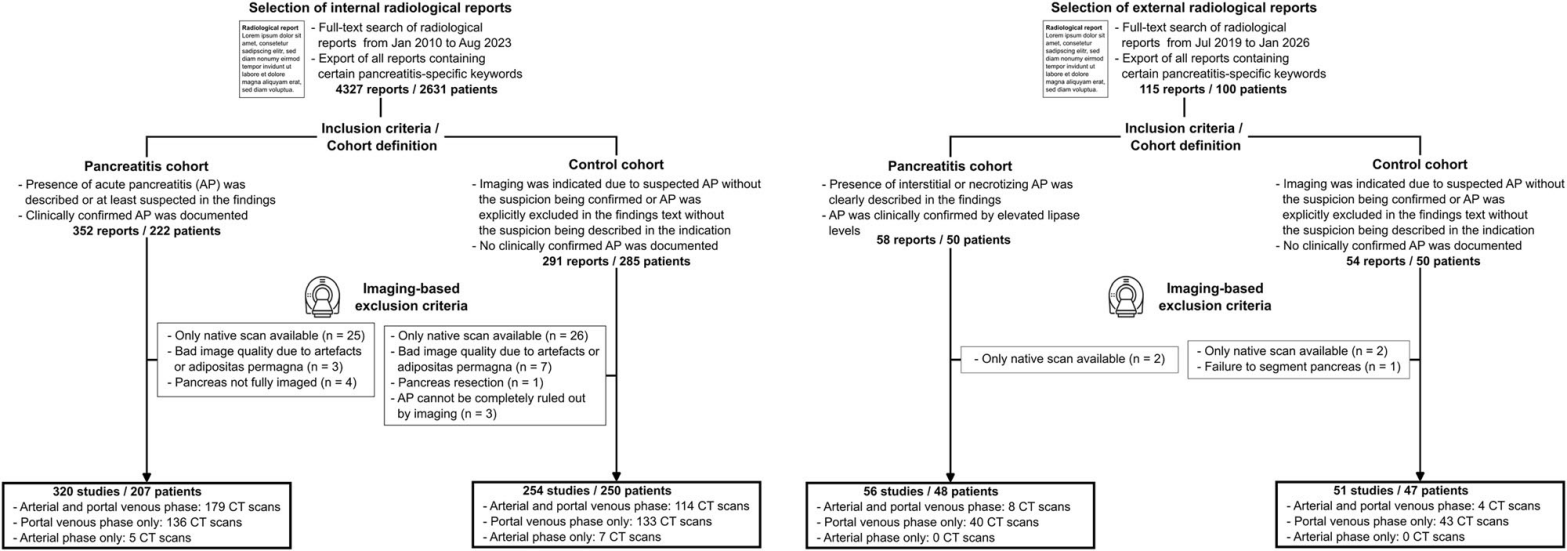
Patient selection flowchart for the creation of the acute pancreatitis and control groups for the internal and external cohorts

Further image-based inclusion criteria were: (1) abdominal CECT in arterial phase (ArP) and/or portal venous phase (PVP); (2) sufficient image quality (exclusion of scans with significant artefacts); and (3) complete pancreatic coverage. CT studies showing prior pancreatic resection, incomplete imaging, or pancreatic malignancy were excluded. A summary of the patient selection process is presented in Fig. 1.

Reference labels (acute pancreatitis *versus* control) were derived retrospectively from the final routine clinical diagnosis, integrating radiological report information with clinical confirmation (*e.g*., ICD-10 coding). This reference standard was not based on an independent blinded re-review of CT images and, therefore, may not be fully independent of the imaging interpretation performed during routine care.

For external validation, CECT scans from 100 patients were retrieved from the Department of Diagnostic and Interventional Radiology, University Medical Center of the Johannes Gutenberg-University, Mainz, Germany, between July 2019 and January 2026. This dataset included 50 patients with AP and 50 control subjects. As for the internal cohort, a full-text search was performed to identify cases with suspected pancreatitis. Patients were included in the AP group if the imaging report clearly described interstitial or necrotizing pancreatitis and the diagnosis was clinically confirmed by elevated lipase levels. Conversely, cases with an initial clinical suspicion of AP that were subsequently ruled out were selected for the control cohort.

### Deep learning-based classification

In a preprocessing step, all CT datasets were cropped to the pancreas region, including the surrounding tissue to account for peripancreatic fat, as described in Supplementary Material S3. The matrix size after cropping the datasets to the pancreas region was defined as 224 × 160 × 160. For image-based detection of AP, different convolutional neural networks based on the ResNet-50 architecture were investigated due to its optimal balance between high performance and computational efficiency for medical image analysis [24]. ResNet-50 was selected as a robust and well-established baseline architecture for volumetric medical image classification in a moderate-sized dataset. The present study was designed to evaluate CT-based AP detection and contrast-phase integration rather than to perform an exhaustive architecture benchmark. Given the limited dataset size, a baseline convolutional neural network was chosen for its strong inductive bias and lower tendency to overfit compared to newer architectures such as Vision Transformers, which require large datasets or specific pretraining strategies to achieve robust performance and generalizability [25]. Cropping to the pancreas region allowed a fully convolutional training in contrast to a patch-wise approach.

The internal dataset was split into training and hold-out test sets, with 20% of both the pancreatitis and control patients randomly selected for the hold-out test set. Partitioning was performed at the patient level to ensure independence between sets. Five-fold cross-validation was used for method development.

**Fig. 2:**
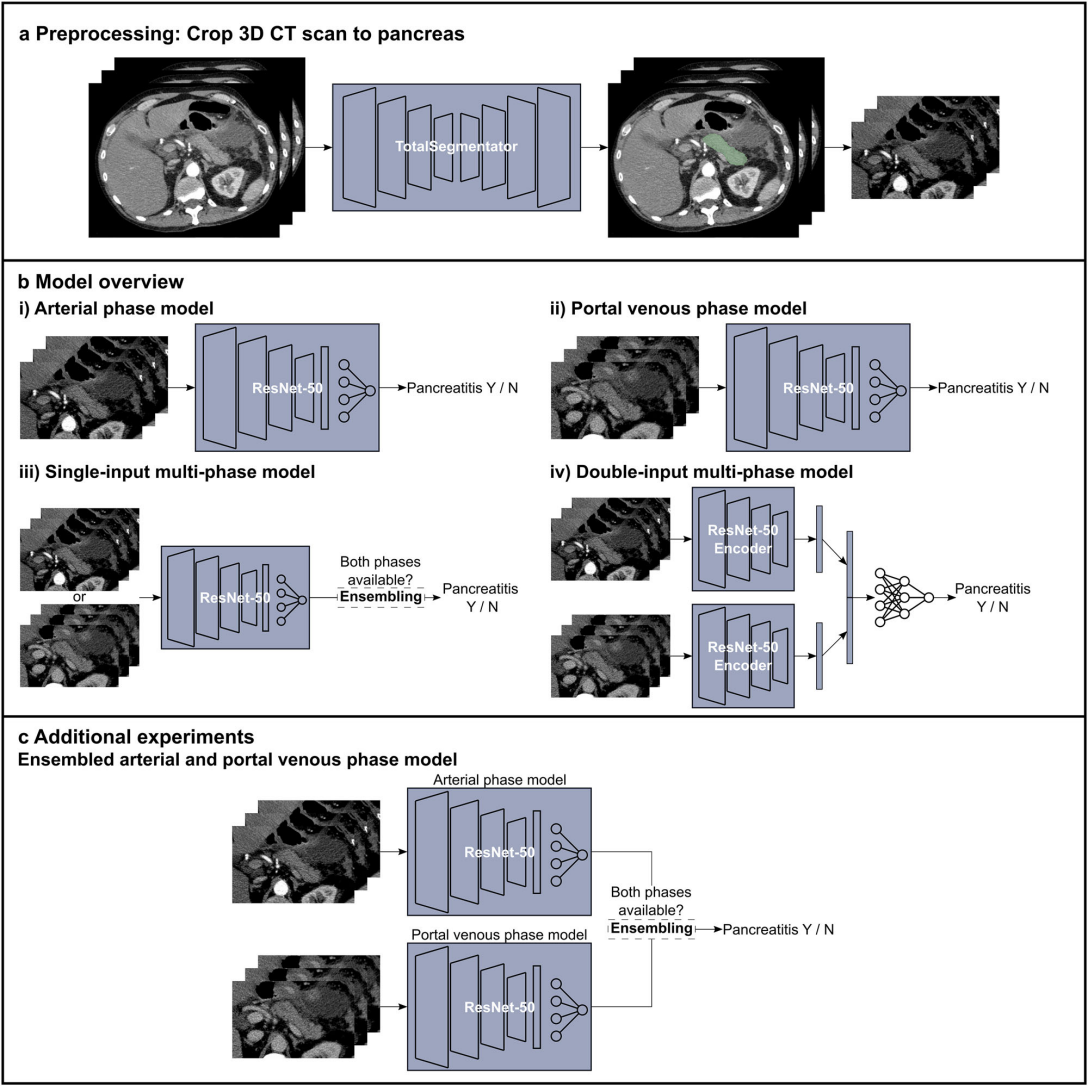
Overview of the methods for the detection of acute pancreatitis from abdominal contrast-enhanced CT. **a** CT datasets were first cropped to the pancreas region based on segmentations obtained with the TotalSegmentator pipeline. **b** In total, four models were developed and evaluated: three single-input models trained with arterial phase, portal venous phase, or both (i–iii), and one double-input model with arterial and portal venous phase as parallel input (iv). **c** In a further experiment, an ensemble of the arterial and the portal venous models was evaluated

A total of four models were trained: First, two separate models were developed, one for the ArP datasets and one for the PVP datasets. Then another model was trained with a single-input channel, but with images of both contrast phases (single-input multiphase model). Prediction probabilities of ArP and PVP were averaged for each patient during inference when both contrast phases were available from the same examination. Finally, a fourth model was trained using both contrast phases as two parallel input channels (double-input multiphase model). This model used a separate ResNet-50 encoder for each contrast phase, whose output was connected after applying an adaptive average pooling followed by three fully connected linear blocks, consisting of a linear layer, ReLU activation, and dropout with a rate of 0.5. Additionally, the combination of the ArP model and the PVP model was investigated (ensembled ArP + PVP model). Details on model training can be found in Supplementary Material S4. An overview of the investigated methods is provided in Fig. 2.

A hyperparameter optimization was performed for the first validation set and selected the best architecture based on the highest F1 score. For the selected model, the hyperparameters were also optimized for the remaining validation splits. Finally, an ensemble of the models from the cross-validation was applied to the internal hold-out and external test set. In a subgroup analysis of the hold-out test set, performance was compared between cases with severe necrotic AP and milder non-necrotic AP (edematous AP), according to the radiological report.

Performance was evaluated using F1 score, accuracy, balanced accuracy, area under the receiver operating characteristic curve (AUROC), precision, recall, and specificity. 95% confidence intervals were determined for all metrics by bootstrapping the internal and external test sets with 1,000 resamples.

## Results

Generation of the internal dataset for training and evaluation resulted in 499 CT scans from 207 patients (age: 53 ± 14 years, mean ± standard deviation, 70 (33.8%) female) in the pancreatitis cohort and 368 CT scans from 250 patients (age: 61 ± 15 years, 110 (44.0%) female) in the control cohort. For the external validation set, 5 out of 100 patients were excluded. Four patients lacked diagnostic contrast-enhancement, and one patient was excluded because automatic pancreas segmentation failed during preprocessing. In total, the final external dataset included 56 CT scans from 48 patients with AP (age: 59 ± 16 years, 16 (33.3%) female; ArP and PVP: 8; PVP only: 40; ArP only: 0) and 51 CT scans from 47 control patients (age: 65 ± 16 years, 25 (53.2%) female; ArP and PVP: 4; PVP only: 43; ArP only: 0).

CT scans were acquired using various CT scanner models (Table 1). Most of the internal scans were also contrasted with an oral contrast agent (*n* = 546, 62% of AP cases and 65% of control cases). Imaging parameters are listed in Table 1. The internal hold-out test set contained 177 CT series from 116 studies from 91 patients (pancreatitis cohort: 100 series, 66 studies, 42 patients, control cohort: 77 series, 50 studies, 49 patients).

**Table 1:** Patient and acquisition characteristics

		AP internal	AP external	Control internal	Control external
Number of cases (*n*)		320	48	254	47
Age (years)		53 ± 14	59 ± 16	61 ± 15	65 ± 16
Sex (female)		70 (33.8%)	16 (33.3%)	110 (44.0%)	25 (53.2%)
Pixel spacing (mm)*		0.80 [0.72, 0.88]	0.84 [0.74, 0.90]	0.79 [0.71, 0.87]	0.76 [0.67, 0.90]
Slice thickness (mm)*		2.0 [1.0, 2.0]	3.0 [3.0, 3.0]	2.0 [1.5, 2.0]	3.0 [3.0, 3.0]
Matrix size*		512 [512, 512]	512 [512, 512]	512 [512, 512]	512 [512, 512]
Tube voltage (kV)*		120 [100, 120]	120 [120, 120]	120 [120, 120]	120 [120, 120]
Scanner					
Philips	Brilliance	96	12	54	15
	iCT	100	32	83	23
	Mx8000 IDT 16	54	-	68	-
Siemens	NAEOTOM Alpha	1	4	4	9
	SOMATOM Force	69	-	45	-
Acquisition phase (*n*)					
Arterial only		5	0	7	0
Portal venous only		136	40	133	43
Both		179	8	114	4

All values are given as mean with standard deviation unless otherwise noted

*AP* Acute pancreatitis, *n* Number of CT scans

* Median with interquartile range

After hyperparameter optimization, the ArP model reached an F1 score of 0.86 (95% confidence interval 0.74–0.95; *n* = 49), and the PVP model an F1 score of 0.91 (0.84–0.97; *n* = 87) on the first validation split. The single-input multiphase model achieved the highest performance with an F1 score of 0.92 (0.84–0.97), considering all studies with either ArP only, PVP only, or both phases (in total *n* = 89). The double-input multiphase model achieved an F1 score of 0.85 only (0.73–0.94; *n* = 47). Ensembling the predictions of the ArP model and the PVP model (when both phases were available) resulted in an F1 score of 0.90 (0.83–0.96), including all (single-phase and dual-phase) datasets (*n* = 89). Details on the grid search results and further performance metrics are listed in Supplementary Material S5.

**Fig. 3:**
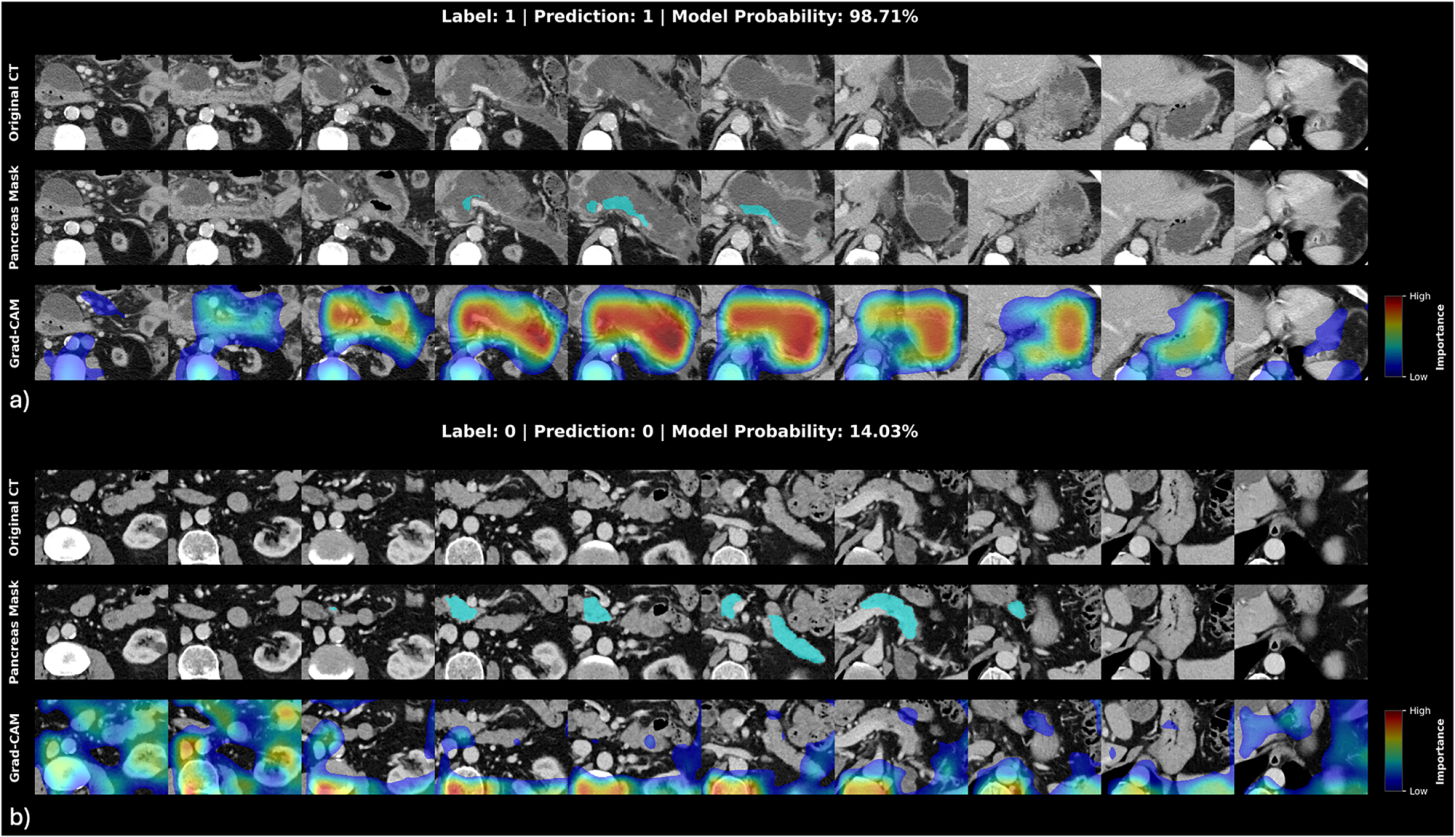
Gradient-weighted class activation mapping (Grad-CAM) overlays for (**a**) a true-positive examination (predicted probability for acute pancreatitis, 98.71%) and (**b**) a true-negative examination (predicted probability, 14.03%). **a** demonstrates marked pancreatic edema, whereas **b** shows no CT evidence of acute pancreatitis. Heatmap intensity indicates the relative contribution of image regions to the model’s prediction (red = higher contribution, blue = lower contribution)

**Fig. 4:**
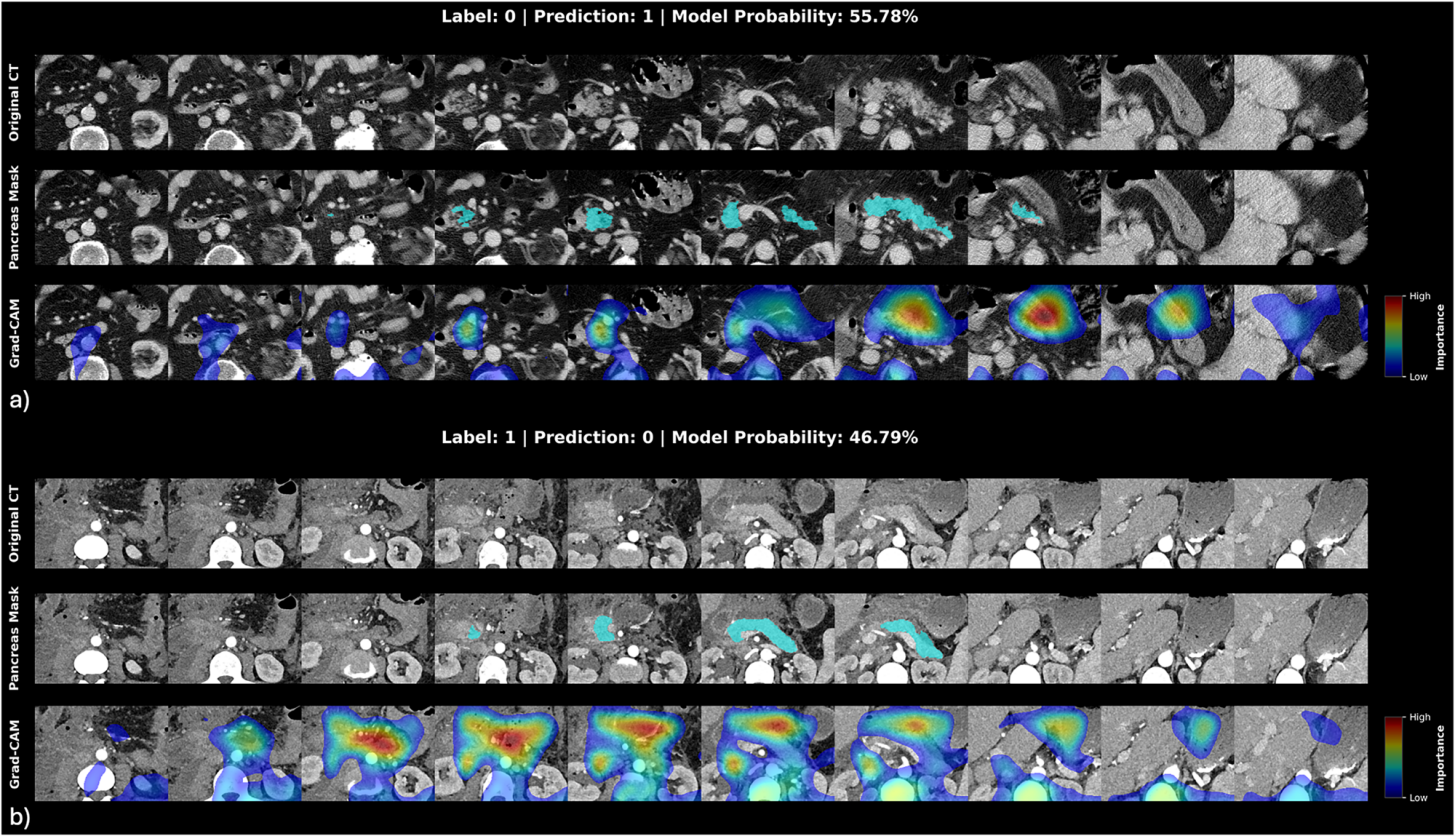
Gradient-weighted class activation mapping (Grad-CAM) overlays for (**a**) a false-positive examination (predicted probability for acute pancreatitis, 55.78%) and (**b**) a false-negative examination (predicted probability, 46.79%). In **a**, the model likely over-weighted lipoatrophic pancreatic changes, resulting in a false-positive classification. In **b**, pancreatic edema contributed less prominently to the prediction, corresponding to a lower estimated likelihood of acute pancreatitis. Heatmap intensity indicates the relative contribution of image regions to the model’s output (red = higher contribution, blue = lower contribution)

Based on these results, the single-input multiphase model was further trained in a 5-fold cross-validated manner, again performing a grid search for each validation fold (Supplementary Material S6). On the hold-out test set (*n* = 116), an F1 score of 0.83 (95% confidence interval 0.75–0.89) was achieved with an AUROC of 0.89 (0.82–0.95). In the internal hold-out test set, 38 studies (57.6%) were noted as edematous AP. For this subset, an F1 score of 0.87 (0.77–0.94) was achieved. Twelve studies (18.2%) in the hold-out test set were marked as AP with pancreatic necrosis, where an F1 score of 0.91 (0.74–1.00) was observed. On the external dataset, the model demonstrated high stability with an AUROC of 0.99 (0.96–1.00) and an F1 score of 0.92 (0.86–0.97). Eighteen studies (32%) in the external hold-out test set were marked as necrotizing AP and 32 (57%) as edematous AP. Additional performance metrics for the internal and external test set are listed in Table 2. Gradient-weighted class activation mapping for selected cases demonstrating true-positive and true-negative cases, as well as false-positive and false-negative cases, which are represented in Figs. 3 and 4, respectively.

**Table 2:** Detailed overview of the single-input multiphase model performance for the detection of acute pancreatitis on the internal hold-out and external test set for every performance metric

	Acc	bAcc	Precision	Recall	F1	Specificity	AUROC
Internal	0.81 [0.73, 0.88]	0.81 [0.73, 0.88]	0.85 [0.77, 0.94]	0.80 [0.70, 0.90]	0.83 [0.75, 0.89]	0.82 [0.70, 0.92]	0.89 [0.82, 0.95]
External	0.92 [0.86, 0.97]	0.92 [0.86, 0.97]	0.87 [0.78, 0.96]	0.98 [0.93, 1.00]	0.92 [0.86, 0.97]	0.85 [0.75, 0.95]	0.99 [0.96, 1.00]

95% confidence intervals in square brackets

*Acc* Accuracy, *bAcc* Balanced accuracy, *AUROC* Area under the receiving operating characteristic curve

The code for using the model can be found at https://github.com/ukb-rad-cfqiai/AP_Detection. Due to legal constraints, the trained model can only be made available after signing a model share agreement, which excludes commercial use.

## Discussion

This work demonstrated the development and evaluation of a DL-based model for the image-based detection of AP on abdominal CECT using data representative of real-world clinical practice. The impact of contrast phase selection was compared using alternative strategies for multiphase integration, with a single-input multiphase model trained on both ArP and PVP images achieving the best results on the internal hold-out test set (AUROC of 0.89, F1 of 0.83). Robustness was further demonstrated in an independent external validation cohort, where performance remained high (AUROC, 0.99; F1 score, 0.92), supporting its generalizability across institutions and underscoring its potential as an adjunctive tool.

Because AP is usually diagnosed from a compatible clinical presentation together with elevated serum lipase, the role of CT, particularly in mild, uncomplicated disease, remains a subject of ongoing debate [26]. In contemporary practice, CECT is most valuable when the diagnosis is uncertain, alternative causes of abdominal pain must be considered, or complications are suspected, including necrosis, peripancreatic collections, hemorrhage, or vascular involvement [9, 26–28]. In these scenarios, CT provides actionable information for risk stratification and management, but image interpretation can vary with reader experience and is susceptible to fatigue-related and cognitive effects such as “satisfaction of search” [29, 30]. As a result, there is an increasing demand for automated diagnostic tools that can aid in the detection and classification of diseases [19, 20, 31, 32].

Advances in AI techniques employing clinical and imaging data have been shown to improve accuracy and efficiency in the diagnosis of various diseases, including AP [21, 31–42]. In the present work, the model was deliberately restricted to imaging input, focusing on CT-based detection of AP from abdominal CECT. To date, only a small body of literature has addressed image-based AP detection on CT [22, 40–42], and among these, only one study specifically evaluated a DL approach for AP detection on CT images. Although Zhang et al described excellent discrimination on an internal hold-out test set (AUROC 0.99), their models generalizability was more modest on external validation (AUROC 0.85), possibly reflecting a development dataset enriched with healthy controls and/or visually overt pancreatitis (*e.g*., marked pancreatic enlargement), which may inflate internal performance while limiting robustness [22]. Our internal cohort was designed to reflect real-world diagnostic uncertainty by including clinically confirmed AP cases with a broad spectrum of imaging features, ranging from overt to subtle or equivocal findings. In contrast, while the external validation cohort also relied on strict clinical confirmation (ICD-10), case selection predominantly required unequivocal radiological signs of AP. This difference in the radiological inclusion threshold represents a classic spectrum effect and likely contributed to the exceptionally high performance (AUROC 0.99) observed in the external set. Our study thus highlights that the performance and clinical transportability of DL models for AP detection are inherently tied to the chosen clinical reference standard and, crucially, to the spectrum of diagnostic certainty in the underlying cohort. Future benchmarking efforts should therefore transparently account for variations in labeling strategies and case selection, alongside institutional heterogeneity.

Prior machine-learning work on AP assessment has largely emphasized radiomics rather than end-to-end DL. Bette et al reported strong discrimination (AUROC 0.93) using radiomics with logistic regression; however, the study was conducted at a single center with largely standardized CT protocols. As the authors noted, radiomics pipelines are particularly sensitive to acquisition factors such as contrast phase selection and protocol parameters, thereby limiting transportability to heterogeneous, less standardized clinical data [41]. Other radiomics studies targeting mild AP have prioritized rapid classification and interpretability, yet achieved only moderate performance (*e.g*., 81% accuracy) and were similarly constrained by limited diversity, including reliance on examinations acquired on a single scanner platform [42]. By contrast, the DL framework evaluated in this work is designed for use on heterogeneous CECT data and may therefore be more compatible with real-world clinical variability. Unlike radiomics, DL does not depend on predefined, hand-engineered descriptors and can learn task-relevant representations directly from images, improving robustness.

The systematic evaluation of strategies for incorporating contrast-phase information in this study was an important developmental step to achieve generalizability due to the vast discrepancy in how examinations are performed between different institutions. We demonstrated that a single-input multiphase model, trained on both ArP and PVP acquisitions, may lead to improved lesion conspicuity, tissue characterization, and vascular assessment, in line with current literature [14, 23]. The lack of incremental benefit with a double-input multiphase architecture likely reflects the reduced effective training sample size, as this approach necessarily relied on the subset of biphasic studies (approximately half of our training cohort). With the broader availability of paired ArP/PVP data, a dedicated double-input design may better exploit inter-phase relationships and could yield additional performance gains.

Several limitations merit consideration. First, the reference standard was based on the final routine clinical diagnosis, which incorporated radiological report findings and clinical information, rather than on an independent, blinded adjudication process. Accordingly, the present study should be interpreted as evaluating image-based classification against a retrospective standard-of-care label, not as a stand-alone diagnostic accuracy study against a fully independent reference standard. Furthermore, despite an emphasis on real-world case selection, the positive class was restricted to examinations in which AP was at least suspected on imaging and subsequently supported by clinical confirmation. Accordingly, we did not include biochemically confirmed AP with initially negative CT findings; future prospective studies with independent blinded multi-reader adjudication and clinically discordant cases are needed.

Second, the model was developed for targeted AP detection in adults undergoing CECT and is not intended as a general acute-abdomen triage tool. This specific definition of the model’s intended use aligns with current recommendations regarding the clinical deployment of AI and the requirements for transparent reporting [43]. Future studies may evaluate performance on non-contrast-enhanced images and the incremental value of incorporating an unenhanced phase when available.

Third, external validation performance was most likely influenced by differences in reference-standard definition: the internal cohort included a substantial proportion of equivocal “suspected” cases, whereas the external cohort included CT findings in addition to biochemical confirmation, resulting in higher recall externally (0.98 *versus* 0.80) with similar specificity (0.85 *versus* 0.82). This highlights spectrum effects and underscores the importance of labeling strategy for reproducibility.

Although transformer-based models are highly promising, they inherently lack the inductive biases of convolutional neural networks and often require substantially larger training datasets or dedicated pretraining strategies to achieve stable generalization without overfitting. Comparative evaluation of newer architectures, including Vision Transformers and hybrid models, under harmonized training conditions will be an important focus of future work.

Additional limitations include the retrospective design and lack of standardized data on symptom-onset–to-CT interval, precluding phase-specific analyses (hyper-acute *versus* later disease). Performance appeared strongest in severe/necrotizing AP, indicating the need for larger datasets enriched for mild disease. Additionally, laboratory or clinical variables were not incorporated, which may improve performance in borderline cases. Although dataset partitioning was strictly performed at the patient level to prevent direct data leakage, the inclusion of repeated examinations may still have introduced within-patient correlation and reduced the effective independence of the dataset, particularly in patients with complicated disease courses. However, the robust diagnostic performance maintained on the independent external validation cohort strongly suggests that the model successfully learned generalizable imaging features rather than overfitting to patient-specific characteristics. Nevertheless, future studies should include one-examination-per-patient sensitivity analyses to explicitly quantify the impact of this effect.

In conclusion, this study presents a DL-based CT classification model for AP with encouraging internal and external performance in retrospectively collected, clinically suspected cases. The results support the feasibility of adjunctive image-based decision support, but prospective evaluation in unselected acute-abdomen populations and against independent adjudication standards is required before clinical deployment.

## Supplementary information

**Additonal File 1 : S1** Full-text search. **S2** List of considered International Statistical Classification of Diseases and Related Health Problems (ICD)-10 codes. **S3** Image preprocessing. **S4** Details on model training. S5 Grid search on the first validation fold. **Table S5.1.** Prediction results for n = 49 arterial phase scans from validation fold 0 using different learning rates (LR) and weight decays (WD). **Table S5.3.** Prediction results for the single input multi-phase model trained on all portal venous and arterial phase scans and evaluated on validation fold 0 (arterial phase only n = 2, portal venous phase only n = 40, both phases n = 47) using different learning rates (LR) and weight decays (WD). **Table S5.4.** Prediction results for the double input multi-phase model trained on biphasic imaging (portal venous and arterial phase scans) and evaluated on validation fold 0 (n = 47) using different learning rates (LR) and weight decays (WD). **Table S5.5** Results for validation fold 0 combining both separate phase models (arterial and portal venous phase model) by ensembling the predictions if biphasic imaging was available. **Table S6.1.** Overview of the validation performance for the selected single input multi-phase model for each validation fold after hyperparameter tuning.

## Data Availability

The datasets generated and analyzed during the current study are not publicly available due to data protection laws.

## Abbreviations

AP: Acute pancreatitis
ArP: Arterial phase
AUROC: Area under the receiver operating characteristic curve
CECT: Contrast-enhanced computed tomography
CT: Computed tomography
DL: Deep learning
ICD-10: International Classification of Diseases, 10th Revision
PVP: Portal venous phase

## Code availability

The code for using the model can be found at https://github.com/ukb-rad-cfqiai/AP_Detection. Due to legal constraints, the trained model can only be made available after signing a model share agreement, which excludes commercial use.
