# Supplementary material for: Deep learning-based detection of acute pancreatitis on abdominal contrast-enhanced CT
Source: Eur Radiol Exp. 2026 Jul 31;10:110. doi: 10.1186/s41747-026-00775-2 (PMC13427702; doi:10.1186/s41747-026-00775-2)
Supplement: Supplementary file 1 — Additonal File 1 : S1 Full-text search. S2 List of considered International Statistical Classification of Diseases and Related Health Problems (ICD)-10 codes. S3 Image preprocessing. S4 Details on model training. S5 Grid search on the first validation fold. Table S5.1. Prediction results for n = 49 arterial phase scans from validation fold 0 using different learning rates (LR) and weight decays (WD). Table S5.3. Prediction results for the single input multi-phase model trained on all portal venous and arterial phase scans and evaluated on validation fold 0 (arterial phase only n = 2, portal venous phase only n = 40, both phases n = 47) using different learning rates (LR) and weight decays (WD). Table S5.4. Prediction results for the double input multi-phase model trained on biphasic imaging (portal venous and arterial phase scans) and evaluated on validation fold 0 (n = 47) using different learning rates (LR) and weight decays (WD). Table S5.5 Results for validation fold 0 combining both separate phase models (arterial and portal venous phase model) by ensembling the predictions if biphasic imaging was available. Table S6.1. Overview of the validation performance for the selected single input multi-phase model for each validation fold after hyperparameter tuning. [file 41747_2026_775_MOESM1_ESM.pdf]

Deep learning-based detection of acute pancreatitis on abdominal contrast-enhanced CT

Electronic Supplementary Material

S1 Full-text search

In the full-text search conducted in the radiology information system between January 2010 and August 2023, all reports were exported containing one of the following keywords in the free text:

- Pankreatitis / Pankreasentzündung / Bauchspeicheldrüsenentzündung (English: pancreatitis)
- Entzündung des Pankreas (English: inflammation of the pancreas)
- entzündliche Veränderung des Pankreas (English: inflammatory change of the pancreas)
- entzündliche Bauchspeicheldrüse / entzündlicher/-s Pankreas (English: inflammatory pancreas)
- ödematöser/-s Pankreas (English: edematous pancreas)
- Verkalkungen des Pankreas (English: calcifications of the pancreas)
- Pseudozyste (English: pseudocyst)
- Pankreasödem (English: pancreatic edema)
- Pankreasverkalkung (English: pancreatic calcification)

S2 List of considered International Statistical Classification of Diseases and Related Health Problems (ICD)-10 codes

The following ICD-10 codes were considered for cohort generation:

- K85.00: Idiopathic acute pancreatitis without necrosis or infection
- K85.01: Idiopathic acute pancreatitis with uninfected necrosis
- K85.10: Biliary acute pancreatitis without necrosis or infection
- K85.11: Biliary acute pancreatitis with uninfected necrosis
- K85.20 Alcohol induced acute pancreatitis without necrosis or infection
- K85.21 Alcohol induced acute pancreatitis with uninfected necrosis
- K85.30 Drug induced acute pancreatitis without necrosis or infection
- K85.31 Drug induced acute pancreatitis with uninfected necrosis
- K85.80: Other acute pancreatitis without necrosis or infection
- K85.81: Other acute pancreatitis with uninfected necrosis
- K85.90: Acute pancreatitis without necrosis or infection, unspecified
- K85.91: Acute pancreatitis with uninfected necrosis, unspecified
- K86.0: Alcohol induced chronic pancreatitis
- K86.1: Other chronic pancreatitis

S3 Image preprocessing

All CT scans were cropped to the pancreas using automated segmentation performed by the deep learning software Total Segmentator (Wasserthal J, Breit HC, Meyer MT, et al (2023) TotalSegmentator: Robust Segmentation of 104 Anatomic Structures in CT Images. Radiol Artif Intell. https://doi.org/10.1148/ryai.230024). Prior to cropping, scans with voxel spacing differing by more than 0.05 mm from the median voxel spacing of the entire dataset were rescaled to match the median. The cropping size was set to the largest expansion of the pancreas in all spatial directions, plus 1 cm, and each crop was centered on the pancreas segmentation. A consistent matrix size was applied to all cropped images, with zero padding used for patients with smaller pancreases. Pixel values were clipped to a range of -400 to 600 Hounsfield units and then linearly normalized to a range of 0 to 1 using min-max scaling.

S4 Details on model training

All experiments were performed in PyTorch (version 2.1.2, Cuda 12.1) with mixed precision on an NVIDIA A100 graphics processing unit with 80 gigabytes of video memory. For all investigated architectures, except the double input multi-phase model, a batch size of 5 with 24 gradient accumulation steps was used. For the double input multi-phase model, a smaller batch size of 2 with 60 gradient accumulation steps was selected.

All models were trained for 10,000 epochs using AdamW optimizer and OneCycle learning rate policy with early stopping after 100 epochs if no improvement was observed on the validation set. For the performed grid search, we investigated the use of different learning rates (10^{-5} to 10^{-3}) and weight decays (0.01 to 0.5).

S5 Grid search on the first validation fold

Arterial phase model

LR	WD	Loss	Epoch	Acc	bAcc	Precision	Recall	F1	Specificity	AUC
10^{-5}	0.01	0.5881	418	0.69	0.66	0.73	0.80	0.76	0.53	0.75
10^{-5}	0.05	0.5877	418	0.69	0.66	0.73	0.80	0.76	0.53	0.75
10^{-5}	0.1	0.5888	418	0.67	0.64	0.71	0.80	0.75	0.47	0.75
10^{-5}	0.5	0.5889	418	0.69	0.66	0.73	0.80	0.76	0.53	0.75
10^{-4}	0.01	0.4232	524	**0.84**	0.83	0.87	**0.87**	**0.87**	0.79	0.89
10^{-4}	0.05	**0.4216**	475	**0.84**	**0.84**	**0.89**	0.83	0.86	**0.84**	0.89
10^{-4}	0.1	0.4241	524	**0.84**	0.83	0.87	**0.87**	**0.87**	0.79	**0.90**
10^{-4}	0.5	0.4269	565	0.82	0.81	0.86	0.83	0.85	0.79	0.87
10^{-3}	0.01	0.5133	20	0.76	0.73	0.78	0.83	0.81	0.63	0.81
10^{-3}	0.05	0.5014	20	0.78	0.76	0.81	0.83	0.82	0.68	0.83
10^{-3}	0.1	0.5062	22	0.78	0.77	0.83	0.80	0.81	0.74	0.82
10^{-3}	0.5	0.5265	20	0.76	0.73	0.78	0.83	0.81	0.63	0.80

Table S5.1: Prediction results for n=49 arterial phase scans from validation fold 0 using different learning rates (LR) and weight decays (WD). Loss refers to the minimum validation loss observed during training and epoch considers the exact epoch number at which this

minimum was reached. Best performance value for each metric is shown in bold and the best hyperparameter setting is highlighted in green.
Acc Accuracy, *bAcc* Balanced accuracy, *AUC* Area under the curve.

Portal venous phase model

LR	WD	Loss	Epoch	Acc	bAcc	Precision	Recall	F1	Specificity	AUC
10^{-5}	0.01	0.3617	1354	0.83	0.83	0.88	0.79	0.83	0.88	0.93
10^{-5}	0.05	0.3847	1204	0.82	0.82	0.90	0.74	0.81	0.90	0.91
10^{-5}	0.1	0.3964	1204	0.83	0.84	**0.94**	0.72	0.82	**0.95**	0.91
10^{-5}	0.5	0.3785	1204	0.82	0.82	0.92	0.72	0.81	0.93	0.92
10^{-4}	0.01	0.3523	553	0.87	0.88	0.93	0.83	0.88	0.93	0.92
10^{-4}	0.05	0.3583	552	**0.91**	**0.91**	0.93	**0.89**	**0.91**	0.93	0.92
10^{-4}	0.1	0.3563	552	0.86	0.86	0.91	0.83	0.87	0.90	0.92
10^{-4}	0.5	0.2966	846	0.85	0.85	0.89	0.83	0.86	0.88	0.95
10^{-3}	0.01	0.2990	443	0.85	0.85	0.90	0.81	0.85	0.90	0.95
10^{-3}	0.05	0.3816	164	0.85	0.85	0.87	0.85	0.86	0.85	0.91
10^{-3}	0.1	**0.2840**	442	0.87	0.88	0.91	0.85	0.88	0.90	**0.97**
10^{-3}	0.5	0.2879	503	0.87	0.88	0.91	0.85	0.88	0.90	0.95

Table S5.2: Prediction results for n=87 portal venouse phase scans from validation fold 0 using different learning rates (LR) and weight decays (WD). Loss refers to the minimum validation loss observed during training and epoch considers the exact epoch number at which this minimum was reached. Best performance value for each metric is shown in bold and the best hyperparameter setting is highlighted in green.
Acc Accuracy, *bAcc* Balanced accuracy, *AUC* Area under the curve.

Single input multi-phase model

LR	WD	Loss	Epoch	Acc	bAcc	Precision	Recall	F1	Specificity	AUC
10^{-5}	0.01	0.3733	979	0.81	0.82	0.92	0.71	0.80	0.93	0.93
10^{-5}	0.05	0.3803	978	0.83	0.83	0.87	0.82	0.84	0.85	0.91
10^{-5}	0.1	0.3871	979	0.82	0.83	0.92	0.73	0.82	0.93	0.91
10^{-5}	0.5	0.3762	978	0.83	0.84	0.89	0.80	0.84	0.88	0.91
10^{-4}	0.01	0.2481	784	0.88	0.88	0.93	0.84	0.88	0.93	**0.97**
10^{-4}	0.05	0.2717	593	0.89	0.89	0.93	0.86	0.89	0.93	0.96
10^{-4}	0.1	**0.2249**	783	**0.91**	**0.91**	0.94	**0.90**	**0.92**	0.93	**0.97**
10^{-4}	0.5	0.2541	784	0.90	0.90	**0.95**	0.86	0.90	**0.95**	0.96
10^{-3}	0.01	0.3054	128	0.87	0.87	0.89	0.86	0.88	0.88	0.95
10^{-3}	0.05	0.2651	489	0.88	0.88	0.90	0.88	0.89	0.88	0.96
10^{-3}	0.1	0.2453	489	0.90	0.90	0.93	0.88	0.91	0.93	**0.97**
10^{-3}	0.5	0.2556	374	0.88	0.88	0.93	0.84	0.88	0.93	0.96

Table S5.3: Prediction results for the single input multi-phase model trained on all portal venous and arterial phase scans and evaluated on validation fold 0 (arterial phase only n=2, portal venous phase only n=40, both phases n=47) using different learning rates (LR) and weight decays (WD). Loss refers to the minimum validation loss observed during training and epoch considers the exact epoch number at which this minimum was reached. Best performance value for each metric is shown in bold and the best hyperparameter setting is highlighted in green.
Acc Accuracy, *bAcc* Balanced accuracy, *AUC* Area under the curve.

Double input multi-phase model

LR	WD	Loss	Epoch	Acc	bAcc	Precision	Recall	F1	Specificity	AUC
10^{-5}	0.01	0.4904	1213	0.83	0.80	0.81	0.93	0.87	0.67	0.87
10^{-5}	0.05	0.499	1213	0.83	0.80	0.81	0.93	0.87	0.67	0.86
10^{-5}	0.1	0.4914	1213	0.83	0.80	0.81	0.93	0.87	0.67	0.87
10^{-5}	0.5	0.4955	1213	0.83	0.80	0.81	0.93	0.87	0.67	0.87
10^{-4}	0.01	0.5024	515	**0.85**	**0.84**	0.86	0.89	**0.88**	0.78	0.89
10^{-4}	0.05	0.5035	515	**0.85**	**0.84**	0.86	0.89	**0.88**	0.78	0.89
10^{-4}	0.1	**0.4628**	621	0.83	**0.84**	**0.92**	0.79	0.85	**0.89**	0.89
10^{-4}	0.5	0.5036	504	**0.85**	0.83	0.84	0.93	**0.88**	0.72	0.88
10^{-3}	0.01	0.5334	217	0.76	0.71	0.74	0.93	0.83	0.50	0.87
10^{-3}	0.05	0.5469	124	0.61	0.50	0.61	**1.00**	0.76	0.00	**0.91**
10^{-3}	0.1	0.5783	99	0.61	0.50	0.61	**1.00**	0.76	0.00	0.79
10^{-3}	0.5	0.5025	124	0.61	0.50	0.61	**1.00**	0.76	0.00	0.92

Table S5.4: Prediction results for the double input multi-phase model trained on biphasic imaging (portal venous and arterial phase scans) and evaluated on validation fold 0 (n=47) using different learning rates (LR) and weight decays (WD). Loss refers to the minimum validation loss observed during training and epoch considers the exact epoch number at which this minimum was reached. Best performance value for each metric is shown in bold and the best hyperparameter setting is highlighted in green.
Acc Accuracy, *bAcc* Balanced accuracy, *AUC* Area under the curve.

Ensembled arterial and portal venous phase model

Acc	bAcc	Precision	Recall	F1	Specificity	AUC
0.90	0.90	0.95	0.86	0.90	0.95	0.92

Table S5.5: Results for validation fold 0 combining both separate phase models (arterial and portal venous phase model) by ensembling the predictions if biphasic imaging was available. Models with optimized hyperparameters were used for both phases.
Acc Accuracy, *bAcc* Balanced accuracy, *AUC* Area under the curve.

S6 Selected model settings after hyperparameter tuning for each validation fold

Fold	LR	WD	Loss	Epoch	Acc	bAcc	Precision	Recall	F1	Specificity	AUC
0	10^{-4}	0.1	0.2249	783	0.91	0.91	0.94	0.90	0.92	0.93	0.97
1	10^{-3}	0.1	0.2141	426	0.90	0.90	0.87	0.96	0.91	0.84	0.98
2	10^{-3}	0.01	0.3215	355	0.89	0.89	0.89	0.89	0.89	0.89	0.95
3	10^{-3}	0.5	0.4451	437	0.80	0.80	0.85	0.81	0.83	0.79	0.88
4	10^{-3}	0.05	0.1805	428	0.94	0.95	0.98	0.92	0.95	0.97	0.98

Table S6.1: Overview of the validation performance for the selected single input multi-phase model for each validation fold after hyperparameter tuning.
LR Learning rate, *WD* Weight decay, *Acc* Accuracy, *bAcc* Balanced accuracy, *AUC* Area under the curve.
